# Effect of magnetic thermal vibration therapy combined with Femoston on endometrial repair in postoperative patients with moderate to severe intrauterine adhesions: a randomized controlled trial

**DOI:** 10.3389/fendo.2026.1851945

**Published:** 2026-08-26

**Authors:** Aiping Wu, Qun Huang, Jing Zhang, Liping Chen

**Affiliations:** Women and Children’s Hospital of Ningbo University, Ningbo, Zhejiang, China

**Keywords:** Femoston therapy, hysteroscopic adhesiolysis, intrauterine adhesions, magnetic thermal vibration therapy, randomized controlled trial, uterine perfusion

## Abstract

**Background:**

Moderate to severe intrauterine adhesions (IUA) remain clinically challenging after hysteroscopic adhesiolysis because endometrial repair may be incomplete, and uterine perfusion may remain suboptimal. This study evaluated whether magnetic thermal vibration therapy (MTVT) combined with Femoston improves postoperative recovery compared with Femoston alone.

**Methods:**

This single-center, parallel-group, randomized controlled superiority trial enrolled 200 postoperative women aged 18–40 years with moderate to severe IUA. Participants were randomized 1:1 to receive Femoston alone (control group) or MTVT plus Femoston (intervention group). The primary outcome was the change in endometrial thickness at 1 month. Secondary outcomes included duration of menstrual bleeding, menstrual blood loss assessed by the Pictorial Blood Loss Assessment Chart (PBAC), Doppler-derived perfusion indices, GQOLI-74 quality-of-life domains, and adverse events. Outcomes were assessed before treatment and 1 month after completion of therapy. Complete-case analysis included 191 participants (control, n=96; intervention, n=95).

**Results:**

Both groups improved after treatment, but the intervention group showed significantly greater improvement in endometrial thickness and uterine perfusion-related parameters. Endometrial thickness increased from 4.7 ± 2.2 to 9.4 ± 3.1 mm in the intervention group versus 4.2 ± 1.9 to 7.9 ± 2.3 mm in the control group (P = 0.013). PBAC scores improved more in the intervention group (30.0 ± 15.3 to 80.7 ± 24.0 vs 31.1 ± 13.8 to 63.8 ± 20.3; P<0.001). Flow index (FI), vascularization index (VI), and vascularization flow index (VFI) increased significantly more, whereas pulsatility index (PI) and resistance index (RI) decreased significantly more, in the intervention group (all P<0.001). Psychological, physical, and social function scores also improved significantly more with the combined regimen, while duration of menstrual bleeding and material-life scores did not differ significantly between groups. Adverse events were uncommon and comparable (9/95 vs 11/96; P = 0.813), and no serious adverse events occurred.

**Conclusion:**

MTVT combined with Femoston may provide greater short-term improvement in endometrial thickness, Doppler-derived perfusion indices, PBAC score, and selected quality-of-life domains compared with Femoston alone in postoperative women with moderate to severe IUA, without an apparent increase in adverse events. However, because this single-center trial primarily assessed short-term surrogate and symptom-based outcomes, larger multicenter studies with longer follow-up and hysteroscopic and reproductive endpoints are needed.

## 1 Introduction

Intrauterine adhesions (IUA), also known as Asherman syndrome, arise after injury to the basal layer of the endometrium and are characterized by fibrosis, cavity distortion, and impaired endometrial regeneration (1–4). Clinically, IUA can present with reduced menstrual flow, amenorrhea, infertility, recurrent pregnancy loss, and other adverse reproductive outcomes, with disease severity closely linked to the degree of cavity damage and the likelihood of postoperative recurrence (1–3, 5, 6).

Hysteroscopic adhesiolysis remains the cornerstone of treatment, particularly for women with moderate to severe disease (1, 3). However, surgery alone does not fully solve the underlying problem of impaired endometrial repair (2, 4, 5). Even after technically successful adhesiolysis, patients remain at risk of incomplete endometrial regeneration, suboptimal uterine perfusion, and re-adhesion, which together may limit menstrual recovery and future fertility (2, 5, 6). Current guidance and recent reviews therefore emphasize the importance of postoperative adjuvant treatment, while also noting that the optimal regimen has not been established (3, 7–10).

Estrogen-based postoperative therapy is widely used because it is intended to stimulate endometrial proliferation and support cavity healing (3, 8). Femoston, a sequential estradiol-dydrogesterone regimen, has been used in this setting, and prior studies have suggested that postoperative hormonal support may improve menstrual recovery and endometrial parameters (7, 11, 12). At the same time, the broader evidence base remains mixed: randomized data have questioned whether hormones alone substantially reduce spontaneous recurrence or improve long-term reproductive outcomes, and recent comparative/meta-analytic work suggests that no single postoperative strategy has emerged as universally superior across all clinically relevant endpoints (9, 10, 13–15).

From the biological perspective, the biological rationale for adjunctive postoperative rehabilitation in IUA is grounded in the pathophysiology of the disease, which involves endometrial fibrosis, inflammation, impaired angiogenesis, and defective regeneration after basal-layer injury. In this context, an intervention capable of improving local microcirculation and supporting tissue repair may theoretically enhance postoperative endometrial recovery beyond hormonal support alone (2, 4, 5). Recent literature has increasingly explored combination postoperative strategies, including mechanical barriers, biologic agents, and device-based or stimulation-based therapies (9, 14–18). Magnetic- and stimulation-based therapies have been investigated in other tissue-repair settings, where they have been associated with modulation of inflammation, angiogenesis, and regenerative signaling, while heat- and vibration-based interventions have been linked to improved local circulation and wound-healing responses. However, the mechanism of MTVT in postoperative IUA has not yet been fully established. For example, electrical stimulation–based approaches have been investigated in postoperative IUA and in thin endometrium, suggesting that physical adjuvant therapies may improve endometrial thickness and related functional markers in selected settings (19–21). However, published evidence specifically evaluating MTVT combined with Femoston after adhesiolysis for moderate to severe IUA remains limited (16, 19–21).

Clinically, in postoperative IUA, improvement in menstrual recovery, endometrial thickness, uterine perfusion, and patient-reported quality of life may reflect more effective restoration of the endometrial microenvironment, especially in women with more advanced adhesions (2, 12, 19, 20, 22). A treatment strategy that can enhance these parameters without increasing adverse events would be of practical importance (9, 14, 15).

Accordingly, this randomized controlled trial was designed to evaluate whether MTVT combined with Femoston provides greater benefit than Femoston alone in postoperative patients with moderate to severe IUA. We hypothesized that the combined regimen would result in greater improvement in endometrial thickness, menstrual recovery, Doppler-based perfusion parameters, and quality-of-life outcomes, while maintaining an acceptable safety profile.

## 2 Methods

### 2.1 Trial design, setting, ethics, and registration

This study was designed as a single-center, parallel-group, randomized controlled superiority trial with a 1:1 allocation ratio. The trial was conducted at Ningbo University Affiliated Women and Children’s Hospital between September 2024 and October 2025. The study protocol was approved by the Medical Ethics Committee of Ningbo University Affiliate Women and Children’s Hospital (approval No. EC2024-125). The study was prospectively registered in the National Medical Research Registration and Filing Information System at medicalresearch.org.cn. (registration No. MR-33-26-043580, date of registration: 2024.09.25). Written informed consent was obtained from all participants before enrollment.

### 2.2 Participants

Eligible participants were postoperative women with moderate to severe intrauterine adhesions (IUA) diagnosed after hysteroscopic evaluation and/or hysteroscopic adhesiolysis (i.e., transcervical resection of adhesions). Baseline disease severity was classified according to the American Fertility Society (AFS) intrauterine adhesion scoring system, with scores of 5–8 considered moderate and scores of 9–12 considered severe (23). Inclusion criterion was postoperative patients with moderate to severe IUA according to the AFS score in the age range of 18–40 years. Exclusion criteria were (1): unstable clinical status, active infection, or concurrent gynecologic disease (2); prior treatment for IUA or recurrent IUA (3); abnormal menstruation attributable to causes other than IUA (4); contraindications to MTVT, estradiol, or dydrogesterone; and (5) severe systemic illness. The trial setting was gynecology ward, and participants were recruited from gynecological surgery clinic.

### 2.3 Randomization, allocation concealment, and blinding

Participants were randomly assigned to the control group or the intervention group in a 1:1 ratio using computer-generated random numbers using R software. Randomization was performed using simple randomization. Allocation concealment was achieved using sequentially numbered, opaque, sealed envelopes. Because the intervention involved active device administration, participants and treating clinicians were unblinded. Outcome assessors, the ultrasonographer, the data manager, and the statistician were blinded.

### 2.4 Interventions

All participants received standard postoperative management after surgery for IUA, including anti-infection treatment and nursing. Participants in the control group received Femoston alone, whereas participants in the intervention group received magnetic thermal vibration therapy (MTVT) combined with Femoston. Femoston was administered according to a standardized institutional postoperative regimen as follows: 2 mg Femoston (estradiol/10 mg dydrogesterone, Abbott Laboratories, USA) on the 1st day after MTVT, and then continued at 2 mg bid for 28 days. MTVT was delivered using Magnetic Vibration and Heat Therapy Unit (CCRZR31, China Care Medical Equipment) according to a standardized institutional protocol. The treatment protocol included disinfecting the device heads using an alcohol swab and placing them on the top of pubic symphysis and the other on the lower hypogastric region. The device has been set to 46°C, with 50mT of magnetic field intensity and 50Hz of vibration frequency and a session duration of 30 minutes each session. The treatment was administered every other day for 20 days, i.e., a total of 10 sessions in treatment course. Because parameter-optimization studies for magnetic thermal vibration therapy in postoperative IUA are currently lacking, the intervention settings were selected according to the device manufacturer’s recommended operating range and the institution’s standardized clinical protocol, with attention to tolerability and procedural consistency across participants and were kept constant for all treated participants. The evidence supporting the plausibility and standardization of the regimen is summarized in **Supplementary Table 1**. The intervention was administered by trained operator, and adherence to the protocol was monitored using session logs and drug counts. No deviations from the planned intervention were recorded.

### 2.5 Outcome measures

On the basis of the proposed mechanism, the intervention was hypothesized to influence short-term endometrial recovery primarily through improvement in local perfusion and tissue-repair conditions, which is why endometrial thickness and Doppler-derived vascular indices were included among the main assessed outcomes.

The prespecified primary outcome was change in endometrial thickness at 1 month, selected as an early surrogate marker of postoperative endometrial recovery. Secondary outcomes included duration of menstruation bleeding, menstrual blood loss assessed by the Pictorial Blood Loss Assessment Chart (PBAC), Doppler-based vascular indices, quality-of-life scores, and adverse events. Assessments were performed before treatment and again at 1 month after completion of therapy, which was selected to evaluate short-term postoperative recovery in menstrual, ultrasound, Doppler, and quality-of-life parameters.

Baseline demographic and clinical characteristics included age, body mass index (BMI), smoking status, alcohol drinking status, and baseline AFS score. Duration of menstruation bleeding was recorded using a menstruation diary. Menstrual blood loss was quantified using the PBAC score. PBAC, originally described in 1990, was modified according to sizes of local coins (24, 25). Endometrial thickness was measured by transvaginal ultrasonography in the sagittal plane around ovulation by the same blinded sonographer. Endometrial and subendometrial perfusion was assessed using Doppler ultrasonography, including flow index (FI), vascularization index (VI), and vascularization flow index (VFI) (26, 27). Uterine artery blood-flow parameters included pulsatility index (PI) and resistance index (RI) were assessed using uterine artery Doppler examination after placing the curvilinear probe on the internal iliac artery and following to uterine arteries. PI and RI were automatically calculated by the device and the values of right and left sides were averaged. Quality of life was assessed using the GQOLI-74, including the domains of material life, psychological function, physical function, and social function (28). Safety outcomes included treatment-emergent adverse events, including allergy, dizziness, breast pain, prolonged abnormal vaginal bleeding, any adverse event, and any serious adverse event. No changes to the trial outcomes were made after enrollment of the first participant.

### 2.6 Sample size calculation

The sample size was calculated on the basis of the prespecified primary outcome, i.e., endometrial thickness. Using a clinically meaningful between-group difference of 0.41 mm, a two-sided α of 0.05, and a power of 80%, the minimum required sample size was estimated to be 95 participants per group (allocation ratio 1/1). Assuming a dropout/loss-to-follow-up rate of 5%, the target enrollment was increased to 200 participants in total. No interim analyses or formal stopping rules were planned.

### 2.7 Statistical analysis

The primary analysis was performed as a complete-case analysis including randomized participants with available 1-month outcome data, analyzed according to their originally assigned groups. Nine randomized participants were lost to follow-up (4 in the control group and 5 in the intervention group) and therefore had missing post-treatment outcome data. No imputation of missing post-treatment outcomes was performed.

Statistical analyses were performed using SPSS version 27. Continuous variables were expressed as mean ± standard deviation (SD), and categorical variables were presented as count (percentage). Baseline between-group comparisons were performed using the independent-samples t test or the Mann-Whitney U test for continuous variables, and the Fisher exact test or chi-square test for categorical variables, as appropriate. Within-group pre- and post-treatment changes were assessed using the paired-samples t test. Between-group differences over time were evaluated using mixed repeated-measures analysis of variance (ANOVA), with group as the between-subject factor and time as the within-subject factor. To further evaluate the independent association between treatment group and outcome improvement, multivariable linear regression models were constructed using the change score for each outcome (Δ = post-treatment value − pre-treatment value) as the dependent variable. Each model was adjusted for the baseline value of the corresponding outcome, AFS IUA score, age, BMI, smoking, and alcohol drinking. Regression coefficients were reported as B values with 95% confidence intervals (95% CIs), together with standardized beta coefficients and adjusted R² values. Because multiple secondary outcomes were evaluated, the corresponding P values were also additionally adjusted using the Benjamini–Hochberg false discovery rate procedure. All tests were two-sided, and P < 0.05 was considered statistically significant.

## 3 Results

### 3.1 Participant flow and recruitment

**Figure 1:**
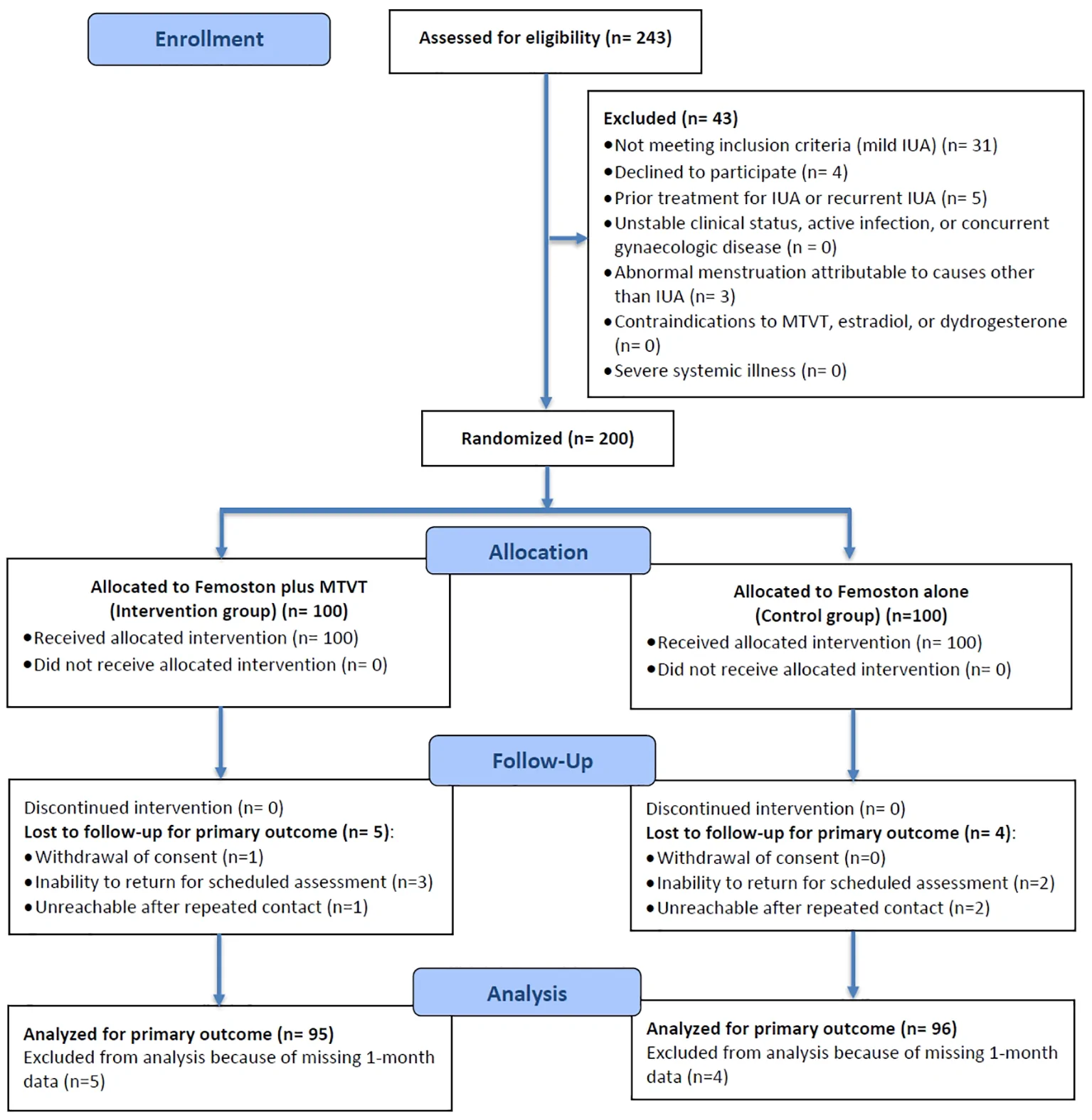
CONSORT participant flow diagram.

Participant recruitment was conducted between September 2024 and September 2025, and all follow-ups were completed by October 2025. A total of 243 postoperative patients were assessed for eligibility. Of these, 43 were excluded before randomization, including 31 who did not meet the inclusion criteria because of mild IUA, 4 who declined to participate, 5 with prior treatment for IUA or recurrent IUA, and 3 with abnormal menstruation attributable to causes other than IUA. The remaining 200 patients with moderate to severe IUA were randomized: 100 were allocated to the control group and 100 to the intervention group. All participants in both groups received the allocated intervention. 100 participants were allocated to the control group and 100 participants were allocated to the intervention group. All participants in the control group and the intervention group started receiving the allocated intervention. During follow-up, 4 participants in the control group and 5 participants in the intervention group were lost to follow-up and excluded from the analysis. The reasons for loss to follow-up were as follows: withdrawal of consent (Control/intervention: 0/1), inability to return for scheduled assessment (Control/intervention:2/3), or unreachable after repeated contact (Control/intervention: 2/1). Losses to follow-up were small and numerically balanced between groups (**Figure 1**) Consequently, 96 participants in the control group and 95 participants in the intervention group were included in the final analysis. The trial ended as planned after the target sample size was reached. The final analyzed sample size was consistent with the planned enrollment target after accounting for losses to follow-up. The primary analysis was performed as a complete-case analysis including randomized participants with available 1-month outcome data, analyzed according to their originally assigned groups. Nine randomized participants were lost to follow-up (4 in the control group and 5 in the intervention group) and therefore had missing post-treatment outcome data.

### 3.2 Baseline characteristics

Baseline demographic and clinical characteristics were generally comparable between the two groups (**Table 1**). The mean age was 32.0 ± 4.9 years in the control group and 32.8 ± 4.6 years in the intervention group. Mean BMI was 23.7 ± 1.3 kg/m² and 24.0 ± 1.9 kg/m², respectively. Smoking and alcohol-drinking status were similar between groups. Baseline AFS score was 8.4 ± 2.1 in the control group and 8.6 ± 2.4 in the intervention group, and the proportion of patients with moderate and severe IUA was also comparable between groups. Baseline duration of menstruation bleeding, PBAC score, and endometrial thickness did not show material between-group imbalance.

**Table 1:** Baseline demographic and clinical characteristics of patients in the control and intervention groups.

Characteristics	Category	Control (n=96)	Intervention (n=95)	P value
Age (years)		32.0 ± 4.9	32.8 ± 4.6	0.277 ^a^
BMI (kg/m²)		23.7 ± 1.3	24.0 ± 1.9	0.263 ^b^
Smoking	Yes	3 (3.1%)	5 (5.3%)	0.497^c^
	No	93 (96.9%)	90 (94.7%)	
Alcohol drinking	Yes	5 (5.2%)	3 (3.2%)	0.721 ^c^
	No	91 (94.8%)	92 (96.8%)	
Intrauterine adhesion severity (AFS score)		8.4 ± 2.1	8.6 ± 2.4	0.556 ^a^
Intrauterine adhesion severity	Moderate (5–8)	49 (51.0%)	43 (45.3%)	0.424 ^d^
	Severe (9–12)	47 (49.0%)	52 (54.7%)	
Duration of menstruation bleeding (days)		3.4 ± 1.1	3.6 ± 1.2	0.395 ^a^
Menstrual blood loss (PBAC score)		31.1 ± 13.8	30.0 ± 15.3	0.591 ^a^
Endometrial thickness (mm)		4.2 ± 1.9	4.7 ± 2.2	0.074 ^a^

^a^ Independent T-test. ^b^Mann-Whitney U-test. ^c^Fisher’s Exact test. ^d^Chi-square test.

### 3.3 Clinical, endometrial, and blood-flow outcomes

Both groups showed significant within-group improvement after treatment in duration of menstruation bleeding, PBAC score, endometrial thickness, FI, VI, VFI, PI, and RI (all within-group P < 0.001; **Table 2**). However, the magnitude of improvement differed between groups for most outcomes. Duration of menstruation bleeding increased from 3.4 ± 1.1 to 4.4 ± 1.3 days in the control group and from 3.6 ± 1.2 to 4.2 ± 1.6 days in the intervention group, with no significant group × time interaction (P = 0.147). In contrast, menstrual blood loss improved more markedly in the intervention group, with the PBAC score increasing from 30.0 ± 15.3 to 80.7 ± 24.0, compared with an increase from 31.1 ± 13.8 to 63.8 ± 20.3 in the control group (P for interaction < 0.001). Endometrial thickness increased from 4.7 ± 2.2 to 9.4 ± 3.1 mm in the intervention group and from 4.2 ± 1.9 to 7.9 ± 2.3 mm in the control group, with a significant between-group difference over time (P = 0.013).

**Table 2:** Changes in menstrual, endometrial, and uterine blood-flow outcomes before and after treatment in the control and intervention groups.

Outcome category	Outcome measure	Control		P within control group ^a^	Intervention		P within intervention group ^a^	P between group ^b^	Adjusted p-value ^c^
		Pre	Post		Pre	Post		P value	
Clinical and imaging parameters	Duration of menstruation bleeding (days)	3.4 ± 1.1	4.4 ± 1.3	<0.001	3.6 ± 1.2	4.2 ± 1.6	<0.001	0.147	0.162
	Menstrual blood loss (PBAC score)	31.1 ± 13.8	63.8 ± 20.3	<0.001	30.0 ± 15.3	80.7 ± 24.0	<0.001	<0.001	0.001
	Endometrial thickness (mm)	4.2 ± 1.9	7.9 ± 2.3	<0.001	4.7 ± 2.2	9.4 ± 3.1	<0.001	0.013	N/A
Endometrial and sub-endometrial blood flow parameters	Flow index (FI)	21.2 ± 5.2	25.2 ± 7.2	<0.001	22.7 ± 5.7	29.5 ± 7.2	<0.001	<0.001	<0.001
	Vascularization index (VI)	3.1 ± 0.9	3.9 ± 1.2	<0.001	3.1 ± 0.8	4.9 ± 1.2	<0.001	<0.001	0.001
	Vascularization flow index (VFI)	0.9 ± 0.3	1.0 ± 0.3	<0.001	0.9 ± 0.2	1.4 ± 0.3	<0.001	<0.001	<0.001
Uterine artery blood flow parameters	Pulsatility Index (PI)	2.1 ± 0.7	1.7 ± 0.5	<0.001	2.0 ± 0.5	1.2 ± 0.3	<0.001	<0.001	<0.001
	Resistance Index (RI)	0.6 ± 0.2	0.5 ± 0.1	<0.001	0.6 ± 0.2	0.4 ± 0.1	<0.001	<0.001	0.001

^a^ Paired T-test. ^b^Mixed repeated-measures ANOVA. ^c^Adjusted using the Benjamini–Hochberg false discovery rate.

Doppler indices also showed greater improvement in the intervention group than in the control group. FI increased from 22.7 ± 5.7 to 29.5 ± 7.2 in the intervention group and from 21.2 ± 5.2 to 25.2 ± 7.2 in the control group (P for interaction < 0.001). VI increased from 3.1 ± 0.8 to 4.9 ± 1.2 in the intervention group and from 3.1 ± 0.9 to 3.9 ± 1.2 in the control group (P < 0.001). VFI increased from 0.9 ± 0.2 to 1.4 ± 0.3 in the intervention group and from 0.9 ± 0.3 to 1.0 ± 0.3 in the control group (P < 0.001). With respect to uterine artery blood flow, PI decreased from 2.0 ± 0.5 to 1.2 ± 0.3 in the intervention group and from 2.1 ± 0.7 to 1.7 ± 0.5 in the control group (P < 0.001), while RI decreased from 0.6 ± 0.2 to 0.4 ± 0.1 in the intervention group and from 0.6 ± 0.2 to 0.5 ± 0.1 in the control group (P < 0.001).

### 3.4 Adverse events

Treatment-emergent adverse events were uncommon and comparable between groups (**Table 3**). In the control group, allergy, dizziness, breast pain, and prolonged abnormal vaginal bleeding occurred in 1, 2, 3, and 5 patients, respectively, compared with 0, 3, 4, and 2 patients in the intervention group. The incidence of any adverse event was 11/96 in the control group and 9/95 in the intervention group (P = 0.813). No serious adverse events were observed in either group. No treatment discontinuation was necessary because of intervention-related side effects.

**Table 3:** Treatment-emergent adverse events in the control and intervention groups.

Side effect	Control	Intervention	P-value ^a^
Allergy	1	0	1
Dizziness	2	3	0.683
Breast pain	3	4	0.721
Prolonged abnormal vaginal bleeding	5	2	0.444
Any adverse event	11	9	0.813
Any serious adverse event	0	0	N/A

^a^ Fisher’s exact test.

### 3.5 Quality-of-life outcomes

Quality-of-life scores improved significantly within both groups across all GQOLI-74 domains (all within-group P < 0.001; **Table 4**). For material life, scores increased from 35.6 ± 7.3 to 41.6 ± 7.0 in the control group and from 36.9 ± 7.5 to 43.3 ± 7.7 in the intervention group, without a significant group × time interaction (P = 0.594). Psychological function improved from 66.2 ± 11.2 to 82.9 ± 10.9 in the control group and from 64.7 ± 11.2 to 84.6 ± 13.4 in the intervention group (P = 0.002). Physical function improved from 71.1 ± 12.5 to 77.8 ± 12.8 in the control group and from 68.0 ± 12.1 to 85.2 ± 12.8 in the intervention group (P < 0.001). Social function improved from 68.0 ± 11.7 to 78.5 ± 12.9 in the control group and from 66.8 ± 11.8 to 86.3 ± 12.7 in the intervention group (P < 0.001).

**Table 4:** Changes in GQOLI-74 domain scores before and after treatment in the control and intervention groups.

Quality-of-life domain	Control		P within control group ^a^	Intervention		P within intervention group ^a^	P between group ^b^	Adjusted p-value ^c^
	Pre	Post		Pre	Post			
Material life (Range 10-50)	35.6 ± 7.3	41.6 ± 7.0	<0.001	36.9 ± 7.5	43.3 ± 7.7	<0.001	0.594	0.594
Psychological function (Range 20-100)	66.2 ± 11.2	82.9 ± 10.9	<0.001	64.7 ± 11.2	84.6 ± 13.4	<0.001	0.002	0.002
Physical function (Range 20-100)	71.1 ± 12.5	77.8 ± 12.8	<0.001	68.0 ± 12.1	85.2 ± 12.8	<0.001	<0.001	0.001
Social function (Range 20-100)	68.0 ± 11.7	78.5 ± 12.9	<0.001	66.8 ± 11.8	86.3 ± 12.7	<0.001	<0.001	0.001

^a^ Paired T-test. ^b^Mixed repeated-measures ANOVA. ^c^Adjusted using the Benjamini–Hochberg false discovery rate.

### 3.6 Adjusted and ancillary analyses

In multivariable linear regression models using change scores as dependent variables, treatment in the intervention group remained significantly associated with greater improvement in several clinical, ultrasound, Doppler, and quality-of-life outcomes after adjustment for baseline outcome value, AFS IUA score, age, BMI, smoking, and alcohol drinking (**Table 5**). Specifically, intervention-group treatment was associated with a greater increase in PBAC score (B = 17.774, 95% CI 12.058 to 23.489; P < 0.001), endometrial thickness (B = 1.227, 95% CI 0.556 to 1.898; P < 0.001), FI (B = 2.765, 95% CI 1.505 to 4.024; P < 0.001), VI (B = 1.063, 95% CI 1.026 to 1.100; P < 0.001), and VFI (B = 0.414, 95% CI 0.402 to 0.425; P < 0.001). Intervention-group treatment was also associated with greater reductions in PI (B = -0.437, 95% CI -0.466 to -0.409; P < 0.001) and RI (B = -0.095, 95% CI -0.100 to -0.089; P < 0.001). By contrast, the change in duration of menstruation bleeding was not significantly associated with treatment group (B = -0.223, 95% CI -0.602 to 0.155; P = 0.246). For quality-of-life outcomes, intervention-group treatment was not significantly associated with the change in material life score (B = 0.715, 95% CI -0.649 to 2.079; P = 0.302), but it was significantly associated with greater improvements in psychological function (B = 2.957, 95% CI 0.891 to 5.024; P = 0.005), physical function (B = 10.298, 95% CI 8.579 to 12.018; P < 0.001), and social function (B = 8.897, 95% CI 7.163 to 10.630; P < 0.001). Exploratory subgroup analysis stratified by baseline adhesion severity (moderate vs severe IUA) showed that the direction of treatment effect was generally consistent across both strata, and no significant group-by-severity interaction was observed for the analyzed outcomes (**Table 6**).

**Table 5:** Multivariable linear regression analysis of the association between treatment group and changes in clinical, imaging, blood-flow, and quality-of-life outcomes.

Δ Outcome (Post-treatment value − pre-treatment value)	B (95% CI)	Standardized B	Adjusted R^2^	P value
Duration of menstruation bleeding	-0.223 (-0.602, 0.155)	-0.079	0.130	0.246
PBAC-score change	17.774 (12.058, 23.489)	0.402	0.198	<0.001
Endometrial thickness	1.227 (0.556, 1.898)	0.242	0.171	<0.001
Flow index (FI)	2.765 (1.505, 4.024)	0.299	0.121	<0.001
Vascularization index (VI)	1.063 (1.026, 1.100)	0.845	0.959	<0.001
Vascularization flow index (VFI)	0.414 (0.402, 0.425)	0.973	0.967	<0.001
Pulsatility Index (PI)	-0.437 (-0.466, -0.409)	-0.759	0.882	<0.001
Resistance Index (RI)	-0.095 (-0.100, -0.089)	-0.646	0.933	<0.001
Material life (Range 10-50)	0.715 (-0.649, 2.079)	0.071	0.118	0.302
Psychological function (Range 20-100)	2.957 (0.891, 5.024)	0.201	0.058	0.005
Physical function (Range 20-100)	10.298 (8.579, 12.018)	0.648	0.447	<0.001
Social function (Range 20-100)	8.897 (7.163, 10.63)	0.596	0.353	<0.001

Δ = post-treatment value − pre-treatment value; models adjusted for baseline outcome value, AFS IUA score, age, BMI, smoking, and alcohol drinking.

**Table 6:** Subgroup analysis of treatment effects stratified by baseline intrauterine adhesion severity (moderate vs severe IUA).

Outcome	Severity subgroup	Δ Control mean ± SD	Δ Intervention mean ± SD	Unadjusted between-group difference (95% CI)	Unadjusted P	Adjusted B (95% CI)	Adjusted P	Interaction P (Group × Severity)
Duration of menstruation bleeding (days)	Moderate	0.76 ± 0.97	0.65 ± 1.48	-0.10 (-0.62 to 0.41)	0.688	-0.14 (-0.64 to 0.36)	0.582	0.696
	Severe	1.06 ± 1.29	0.58 ± 1.75	-0.49 (-1.11 to 0.13)	0.122	-0.25 (-0.83 to 0.34)	0.404	0.696
Menstrual blood loss (PBAC score)	Moderate	32.82 ± 14.14	50.07 ± 24.73	17.25 (9.03 to 25.47)	<0.001	17.26 (8.79 to 25.72)	<0.001	0.637
	Severe	32.49 ± 14.25	51.31 ± 25.49	18.82 (10.46 to 27.18)	<0.001	19.40 (11.38 to 27.43)	<0.001	0.637
Endometrial thickness (mm)	Moderate	3.51 ± 2.77	4.44 ± 1.96	0.92 (-0.08 to 1.93)	0.072	1.09 (0.17 to 2.01)	0.021	0.600
	Severe	3.95 ± 2.83	4.81 ± 2.33	0.86 (-0.17 to 1.89)	0.102	1.49 (0.48 to 2.50)	0.004	0.600
Flow index (FI)	Moderate	4.14 ± 5.72	6.51 ± 2.00	2.37 (0.54 to 4.19)	0.012	2.26 (0.42 to 4.10)	0.017	0.510
	Severe	3.79 ± 6.26	7.04 ± 1.62	3.25 (1.46 to 5.04)	<0.001	3.13 (1.31 to 4.94)	<0.001	0.510
Vascularization index (VI)	Moderate	0.81 ± 0.24	1.82 ± 0.46	1.01 (0.86 to 1.16)	<0.001	1.06 (1.00 to 1.11)	<0.001	0.857
	Severe	0.79 ± 0.25	1.87 ± 0.41	1.08 (0.95 to 1.22)	<0.001	1.07 (1.02 to 1.12)	<0.001	0.857
Vascularization flow index (VFI)	Moderate	0.11 ± 0.03	0.50 ± 0.11	0.39 (0.36 to 0.42)	<0.001	0.41 (0.40 to 0.43)	<0.001	0.798
	Severe	0.11 ± 0.03	0.52 ± 0.09	0.41 (0.38 to 0.43)	<0.001	0.42 (0.40 to 0.43)	<0.001	0.798
Pulsatility index (PI)	Moderate	-0.40 ± 0.18	-0.77 ± 0.26	-0.37 (-0.46 to -0.28)	<0.001	-0.44 (-0.48 to -0.40)	<0.001	0.919
	Severe	-0.39 ± 0.17	-0.80 ± 0.24	-0.40 (-0.49 to -0.32)	<0.001	-0.44 (-0.48 to -0.40)	<0.001	0.919
Resistance index (RI)	Moderate	-0.05 ± 0.04	-0.14 ± 0.08	-0.08 (-0.11 to -0.06)	<0.001	-0.09 (-0.10 to -0.09)	<0.001	0.850
	Severe	-0.05 ± 0.04	-0.14 ± 0.07	-0.09 (-0.12 to -0.07)	<0.001	-0.10 (-0.10 to -0.09)	<0.001	0.850
Material life	Moderate	5.49 ± 4.76	6.67 ± 5.23	1.18 (-0.88 to 3.25)	0.258	1.09 (-0.85 to 3.03)	0.267	0.493
	Severe	6.47 ± 4.73	6.10 ± 5.43	-0.37 (-2.41 to 1.67)	0.718	0.15 (-1.87 to 2.17)	0.883	0.493
Psychological function	Moderate	15.78 ± 7.60	19.00 ± 6.97	3.22 (0.19 to 6.26)	0.038	3.02 (-0.08 to 6.13)	0.056	0.936
	Severe	17.62 ± 8.24	20.65 ± 5.93	3.04 (0.19 to 5.88)	0.037	2.54 (-0.34 to 5.42)	0.083	0.936
Physical function	Moderate	5.78 ± 4.95	16.21 ± 7.18	10.43 (7.90 to 12.96)	<0.001	9.87 (7.35 to 12.38)	<0.001	0.862
	Severe	7.66 ± 5.18	18.04 ± 6.34	10.38 (8.06 to 12.70)	<0.001	10.51 (8.09 to 12.94)	<0.001	0.862
Social function	Moderate	9.69 ± 4.72	18.72 ± 7.03	9.03 (6.57 to 11.48)	<0.001	8.78 (6.31 to 11.25)	<0.001	0.967
	Severe	11.34 ± 5.49	20.08 ± 6.50	8.74 (6.32 to 11.15)	<0.001	9.02 (6.51 to 11.53)	<0.001	0.967

Δ = post-treatment value − pre-treatment value. Unadjusted results are based on between-group comparisons of change scores within each severity subgroup. Adjusted results are from linear regression models within each subgroup, adjusted for the baseline value of the corresponding outcome, age, BMI, smoking, and alcohol drinking. Interaction P values test the group × severity interaction in the overall dataset. Subgroup analyses were exploratory and not prespecified or powered for formal subgroup inference.

## 4 Discussion

In this randomized controlled trial, postoperative patients with moderate to severe intrauterine adhesions who received MTVT combined with Femoston showed greater improvement than those who received Femoston alone in PBAC score, endometrial thickness, FI, VI, VFI, PI, and RI. These associations remained significant after multivariable adjustment, and the intervention was also associated with greater improvement in psychological, physical, and social functioning, whereas duration of menstrual bleeding and material-life score did not differ significantly between groups. The adjusted between-group effect on endometrial thickness was modest in absolute terms (approximately 1.23 mm), whereas the difference in PBAC improvement (17.77 points) may be more directly interpretable clinically. Adverse-event rates were low and comparable between groups, and no serious adverse events were observed. Overall, these findings suggest that the combined regimen may enhance endometrial recovery and uterine perfusion without compromising safety.

The primary endpoint in the present trial was endometrial thickness at 1 month, which should be interpreted as an early surrogate marker rather than a definitive clinical outcome. Although endometrial thickness is commonly used as an indicator of postoperative endometrial recovery, it does not by itself reliably establish reduced adhesion recurrence, improved fertility, or live birth benefit. More clinically meaningful long-term outcomes include second-look hysteroscopic findings, readhesion/recurrence rate, pregnancy, and live birth, none of which were assessed in the current study. Therefore, our results should be interpreted as evidence of short-term structural and perfusion-related improvement, not proof of long-term reproductive benefit.

The present findings are broadly consistent with the established concept that postoperative adjuvant therapy is important after hysteroscopic adhesiolysis, particularly in women with moderate or severe disease, in whom recurrence and incomplete endometrial recovery remain major clinical challenges (3, 5, 14). Current guidance identifies hysteroscopic treatment as the cornerstone of management, but also recognizes that no single postoperative strategy has yet emerged as clearly superior across all clinically meaningful endpoints (3, 5, 14). More recent evidence syntheses likewise show an active but still unsettled field, with multiple barriers, hormonal regimens, and biologic interventions under evaluation (5, 14, 16).

Our results also align with prior studies suggesting that hormone-based postoperative management can improve menstruation and endometrial recovery, but they further suggest that adding a physical adjunct may enhance this response (7, 10, 12, 13, 29). In a clinical study of severe IUA, Lan et al. reported that treatment involving Femoston was associated with improved menstruation and endometrial recovery, supporting the general role of postoperative estrogen-progestin support in this setting (29). Other randomized studies have examined how postoperative estrogen is delivered; e.g., Guo et al. compared estrogen doses after adhesiolysis, and Yi et al. found that transdermal estrogen was similarly efficacious to oral therapy for menstruation, endometrial thickness, and fertility-related outcomes (7, 12). At the same time, Hanstede et al. reported that hormonal support alone did not lead to better outcomes in women with Asherman syndrome, underscoring that hormones by themselves may be insufficient for all patients (10, 13). Against that background, our results are notable because the intervention effect persisted even after multivariable adjustment, suggesting that the added improvement in endometrial thickness, Doppler, and quality of life indices may not be explained simply by baseline imbalances.

A particularly interesting aspect of this study is the consistent improvement in Doppler-derived vascular indices. Compared with Femoston alone, the combined regimen produced larger gains in FI, VI, and VFI and larger reductions in PI and RI, which together support the possibility of a more favorable postoperative uterine perfusion or microcirculatory environment. However, these Doppler-derived measures are indirect vascular markers and should not be interpreted as direct evidence of improved uterine receptivity. This pattern is directionally consistent with other postoperative IUA literature in which adjunctive therapies targeting endometrial repair have been associated with better endometrial thickness, adhesion-related outcomes, or blood-flow-related parameters (16, 30, 31). Experimental and translational studies also support the biologic plausibility that endometrial repair in IUA is linked to fibrosis regulation, extracellular-matrix remodeling, angiogenesis, and inflammatory signaling, providing a rationale for interventions that may improve local tissue perfusion and remodeling.

The hypothesized biological interpretation of the present findings can connect the intervention with observed outcomes. Intrauterine adhesions are characterized by fibrotic repair, inflammation, impaired angiogenesis, and defective endometrial regeneration, providing a plausible rationale for adjunctive therapies that may improve local perfusion and tissue-healing conditions. Experimental and clinical literature outside the immediate IUA setting suggests that pulsed electromagnetic stimulation may influence inflammation, angiogenesis, and regenerative signaling, whereas heat and vibration therapies have been associated with improved local circulation and wound-healing responses (2, 4, 16). On this basis, the MTVT used in the present trial may plausibly contribute to improved short-term endometrial thickness and Doppler indices. However, because direct mechanistic evidence in postoperative IUA is limited, the present study cannot determine which component of the intervention is responsible for the observed effect, nor can it establish the precise biological pathway involved.

The present study also adds a patient-reported dimension that is less commonly emphasized in postoperative IUA trials (3, 5, 14, 22). In addition to objective ultrasound and Doppler improvements, the intervention group showed significantly greater gains in psychological, physical, and social functioning, whereas improvement in material life did not differ significantly between groups. This pattern is clinically plausible: improvements in menstrual recovery, symptom burden, and perceived uterine recovery may reasonably translate into better psychosocial and functional well-being, while material-life scores may be less sensitive to short-term therapeutic change (2, 22). At the same time, the current IUA literature has focused much more heavily on menstrual parameters, endometrial thickness, AFS scores, recurrence, and fertility than on quality-of-life outcomes, so this part of the study may represent a useful extension of the evidence base (2, 3, 5, 14).

Another strength of this study is that the primary efficacy signal was not limited to simple unadjusted pre-post comparisons. The between-group advantage remained evident in multivariable regression models that accounted for baseline outcome values, AFS score, age, BMI, smoking, and alcohol drinking, which supports the robustness of the observed differences. In addition, the baseline distribution of moderate and severe IUA was comparable between groups, reducing concern that the main findings were driven by an obvious initial disease-severity imbalance. The randomized parallel-group design, blinded outcome assessment, and consistent 1-month postoperative evaluation also strengthen the internal coherence of the trial.

Several internal validity issues should be considered when interpreting the present findings. First, participants and treating clinicians were not blinded because of the nature of the intervention. This may have introduced performance or reporting bias, particularly for subjective outcomes such as PBAC-based menstrual blood-loss assessment and GQOLI-74 quality-of-life scores. By contrast, the primary ultrasound and Doppler-based outcomes were assessed by blinded personnel and are less likely to have been influenced by a lack of participant blinding. Second, the primary analysis was conducted as a complete-case analysis because 9 randomized participants were lost to follow-up, and no missing-data imputation was performed. Although the number of losses was small and balanced between groups, this approach does not constitute a full intention-to-treat analysis and may have introduced some degree of attrition bias.

Nevertheless, the study has several important limitations. First, this was a single-center trial conducted at a tertiary women’s hospital and enrolled a relatively selected population of postoperative women aged 18–40 years with moderate to severe IUA, excluding patients with recurrent IUA, major comorbid gynecologic disease, and severe systemic illness. Therefore, the findings may not be directly generalizable to women with mild adhesions, recurrent IUA, different age groups, non-postoperative populations, or patients managed in different centers. Second, the follow-up period was limited to 1 month, so the findings primarily reflect short-term recovery. Although endometrial thickness, PBAC score, and Doppler-derived vascular indices are meaningful short-term markers, they do not substitute for second-look hysteroscopy, adhesion recurrence/readhesion rate, sustained menstrual normalization, clinical pregnancy, or live birth, which are among the most important patient-centered outcomes in IUA research (2, 3, 5, 14). The current evidence landscape suggests that short-term improvements in endometrial or AFS-related measures do not always translate into clear differences in pregnancy outcomes across interventions (5, 13, 14, 30). For example, Shen et al. found that PRP improved the reduction in adhesion score at second-look hysteroscopy, but the recurrence-rate difference was not statistically significant (32), and the 2024 network meta-analysis found no clear between-intervention difference in clinical pregnancy despite favorable rankings for some combinations (5, 14, 33). Third, although Doppler indices provide useful information on uterine and endometrial perfusion, they are indirect markers and should not be overinterpreted as direct evidence of uterine receptivity or future reproductive success. Fourth, participants and treating clinicians were unblinded because of the nature of the intervention, which may have introduced bias in subjective outcomes, particularly PBAC and quality-of-life measures, although ultrasound and Doppler outcomes were assessed by blinded personnel. Fifth, the primary analysis was conducted as a complete-case analysis because 9 randomized participants were lost to follow-up and no imputation of missing outcomes was performed; although the number of losses was small and balanced between groups, this may have introduced some degree of attrition bias. Finally, the biological mechanism of MTVT in postoperative IUA is not yet fully established. Although the intervention has a plausible rationale based on tissue-repair and microcirculatory principles, direct uterine mechanistic evidence and parameter-optimization studies remain limited. Considering these limitations, future trials should include multi-center recruitment, incorporate longer follow-up, second-look hysteroscopy, standardized recurrence outcomes, and reproductive endpoints such as clinical pregnancy and live birth (3, 5, 14). Nevertheless, it would be valuable to determine in longer-term follow-up whether the observed improvements in endometrial thickness and perfusion are linked to better cavity healing and subsequent reproductive outcomes. Although exploratory subgroup analyses did not show clear evidence of treatment-effect heterogeneity between moderate and severe IUA, the trial was not powered for formal subgroup inference, and future studies should evaluate severity-specific treatment effects in larger cohorts.

## 5 Conclusion

In conclusion, this randomized trial suggests that MTVT combined with Femoston may provide greater short-term improvement in endometrial thickness, Doppler indices, PBAC score, and selected quality-of-life domains than Femoston alone for postoperative women with moderate to severe intrauterine adhesions, particularly with respect to menstrual blood loss, endometrial thickness, uterine perfusion indices, and several quality-of-life domains, without an apparent increase in adverse events. These findings support further investigation of the combined regimen as a postoperative adjuvant strategy. However, because this single-center study primarily assessed short-term surrogate and symptom-based outcomes, larger multicenter trials with hysteroscopic, recurrence, and reproductive endpoints are needed for assessment of long-term clinical benefit.

## Data Availability

The original contributions presented in the study are included in the article/**Supplementary Material**. Further inquiries can be directed to the corresponding author.

## References

[1] Dreisler E Kjer JJ . Asherman's syndrome: current perspectives on diagnosis and management. Int J Womens Health. (2019) 11:191–8. doi: 10.2147/IJWH.S165474 PMC6430995 30936754

[2] Hooker AB de Leeuw RA Emanuel MH Mijatovic V Brolmann HAM Huirne JAF . The link between intrauterine adhesions and impaired reproductive performance: a systematic review of the literature. BMC Pregnancy Childbirth. (2022) 22:837. doi: 10.1186/s12884-022-05164-2 36376829 PMC9664654

[3] Surgery AEG . AAGL practice report: practice guidelines on intrauterine adhesions developed in collaboration with the European Society of Gynaecological Endoscopy (ESGE). Gynecol Surg. (2017) 14:6. doi: 10.1186/s10397-017-1007-3 28603474 PMC5440524

[4] Zhao G Hu Y . Mechanistic insights into intrauterine adhesions. Semin Immunopathol. (2024) 47:3. doi: 10.1007/s00281-024-01030-9 39613882

[5] Munro MG Salazar CA Bhagavath B Emanuel MH Huddleston HG Sobti D . The epidemiology, clinical burden, and prevention of intrauterine adhesions (IUAs) related to surgically induced endometrial trauma: a systematic literature review and selective meta-analyses. Hum Reprod Update. (2025) 31:588–625. doi: 10.1093/humupd/dmaf019 40914965 PMC12584913

[6] Wang L Guo C Cao H . Effect of hysteroscopic adhesiolysis on recurrence, menstruation and pregnancy outcomes in patients with different degrees of intrauterine adhesions. Am J Transl Res. (2022) 14:484–90. PMC8829641 35173868

[7] Guo J Li TC Liu Y Xia E Xiao Y Zhou F . A prospective, randomized, controlled trial comparing two doses of oestrogen therapy after hysteroscopic adhesiolysis to prevent intrauterine adhesion recurrence. Reprod BioMed Online. (2017) 35:555–61. doi: 10.1016/j.rbmo.2017.07.011 28784336

[8] Johary J Xue M Zhu X Xu D Velu PP . Efficacy of estrogen therapy in patients with intrauterine adhesions: systematic review. J Minim Invasive Gynecol. (2014) 21:44–54. doi: 10.1016/j.jmig.2013.07.018 23933351

[9] Yan Y Xu D . The effect of adjuvant treatment to prevent and treat intrauterine adhesions: a network meta-analysis of randomized controlled trials. J Minim Invasive Gynecol. (2018) 25:589–99. doi: 10.1016/j.jmig.2017.09.006 28893657

[10] Yang L Ma N Song D Huang X Zhou Q Guo Y . The effect of estrogen in the prevention of adhesion reformation after hysteroscopic adhesiolysis: a prospective randomized control trial. J Minim Invasive Gynecol. (2022) 29:871–8. doi: 10.1097/ogx.0000000000001066 35439645

[11] Sun Y Chen X Qian Z Cao L Zhan S Huang L . Estradiol and intrauterine device treatment for moderate and severe intrauterine adhesions after transcervical resection. BMC Womens Health. (2022) 22:357. doi: 10.1186/s12905-022-01940-6 36038909 PMC9422139

[12] Yi T Zhang X Gupta V Li L Zhong Q . Transdermal estrogen gel vs oral estrogen after hysteroscopy for intrauterine adhesion separation: a prospective randomized study. Front Endocrinol (Lausanne). (2023) 14:1066210. doi: 10.3389/fendo.2023.1066210 36967790 PMC10031037

[13] Hanstede MMF van Stralen KJ Molkenboer JFM Veersema S Emanuel MH . Hormonal support in women with Asherman syndrome does not lead to better outcomes: a randomized trial. Reprod Med Biol. (2023) 22:e12526. doi: 10.1002/rmb2.12526 37396823 PMC10308500

[14] Tang R Zhang W Xiao X Li W Chen X Wang X . Intrauterine interventions options for preventing recurrence after hysteroscopic adhesiolysis: a systematic review and network meta-analysis of randomized controlled trials. Arch Gynecol Obstet. (2024) 309:1847–61. doi: 10.1007/s00404-024-07460-y 38493418

[15] Vitale SG Riemma G Carugno J Perez-Medina T Alonso Pacheco L Haimovich S . Postsurgical barrier strategies to avoid the recurrence of intrauterine adhesion formation after hysteroscopic adhesiolysis: a network meta-analysis of randomized controlled trials. Am J Obstet Gynecol. (2022) 226:487–98:e8. doi: 10.1016/j.ajog.2021.09.015 34555319

[16] Ma J Zhan H Li W Zhang L Yun F Wu R . Recent trends in therapeutic strategies for repairing endometrial tissue in intrauterine adhesion. Biomater Res. (2021) 25:40. doi: 10.1186/s40824-021-00242-6 34819167 PMC8611984

[17] Pan LZ Wang Y Chen X . A randomized controlled study on an integrated approach to prevent and treat re-adhesion after transcervical resection of moderate-to-severe intrauterine adhesions. Clinics (Sao Paulo). (2021) 76:e1987. doi: 10.6061/clinics/2021/e1987 33978070 PMC8075111

[18] Feng L Zhu B Liu Z Jia R Chen Y Ren Y . A versatile approach to synergistically combine physical barriers and bioactive agents for intrauterine adhesion prevention. Adv Funct Mater. (2026) 36:e14793. doi: 10.1002/adfm.202514793 41531421

[19] Bodombossou-Djobo MM Zheng C Chen S Yang D . Neuromuscular electrical stimulation and biofeedback therapy may improve endometrial growth for patients with thin endometrium during frozen-thawed embryo transfer: a preliminary report. Reprod Biol Endocrinol. (2011) 9:122. doi: 10.1186/1477-7827-9-122 21867532 PMC3180360

[20] Shabiti Y Wufuer S Tuohuti R Yun T Lu J . Impact of biomimetic electrical stimulation combined with Femoston on pregnancy rate and endometrium characteristics in infertility patients with thin endometrium: a prospective observational study. Gynecol Endocrinol. (2023) 39:2214629. doi: 10.1080/09513590.2023.2214629 37204000

[21] Xu T Xie L Qin X Xie X Mo S Jiang Q . Efficacy of electrical stimulation combined with ultrasound acupuncture therapy on treatment in patients with intrauterine adhesions: study protocol for a placebo-controlled, single-blind, single-center, randomized trial. Med (Baltimore). (2022) 101:e31469. doi: 10.1097/md.0000000000031469 36550829 PMC9771222

[22] Wang PH Yang ST Chang WH Liu CH Liu HH Lee WL . Intrauterine adhesion. Taiwanese J Obstetrics Gynecology. (2024) 63:312–9. doi: 10.1016/j.tjog.2024.02.004 38802193

[23] The American Fertility Society . The American Fertility Society classifications of adnexal adhesions, distal tubal occlusion, tubal occlusion secondary to tubal ligation, tubal pregnancies, mullerian anomalies and intrauterine adhesions. Fertil Steril. (1988) 49:944–55. doi: 10.1159/000401246 3371491

[24] Higham JM O'Brien PM Shaw RW . Assessment of menstrual blood loss using a pictorial chart. Br J Obstetrics Gynaecology. (1990) 97:734–9. doi: 10.1111/j.1471-0528.1990.tb16249.x 2400752

[25] Ko JKY Lao TT Cheung VYT . Pictorial Blood Loss Assessment Chart for evaluating heavy menstrual bleeding in Asian women. Hong Kong Med J = Xianggang Yi Xue Za Zhi. (2021) 27:399–404. doi: 10.12809/hkmj208743 34949729

[26] Alcazar JL . Three-dimensional power Doppler derived vascular indices: what are we measuring and how are we doing it? Ultrasound Obstet Gynecol. (2008) 32:485–7. doi: 10.1002/uog.6144 18726929

[27] Sadek S Matitashvili T Kovac A Ramadan H Stadtmauer L . Assessment of uterine receptivity by endometrial and sub-endometrial blood flow using SlowflowHD in hormone prepared frozen embryo transfer cycles: a pilot study. J Assisted Reprod Genet. (2022) 39:1069–79. doi: 10.1007/s10815-022-02454-8 35426062 PMC9107536

[28] Zheng Y Lei F Liu B . Cancer diagnosis disclosure and quality of life in elderly cancer patients. Healthcare [Internet]. (2019) 7:163. doi: 10.3390/healthcare7040163 31847309 PMC6956195

[29] Yi Lan CR Yugang C Boping Y Liyuan S Li W . Effect of transcervical resection of adhesion combined with femoston on endometrium and MMP-9 levels in patients with severe intrauterine adhesions. Int J Clin Exp Med. (2020) 13:7.

[30] Chen Y Liu L Luo Y Chen M Huan Y Fang R . Effects of aspirin and intrauterine balloon on endometrial repair and reproductive prognosis in patients with severe intrauterine adhesion: a prospective cohort study. BioMed Res Int. (2017) 2017:8526104. doi: 10.1155/2017/8526104 28251159 PMC5303840

[31] Li LN Li XD Du J . The effect of aspirin on uterine arterial blood flow and endometrium in moderate and severe intrauterine adhesion after transcervical resection of adhesion: a systematic review and meta-analysis. J Matern Fetal Neonatal Med. (2023) 36:2209818. doi: 10.1080/14767058.2023.2209818 37286223

[32] Shen M Duan H Lv R Lv C . Efficacy of autologous platelet-rich plasma in preventing adhesion reformation following hysteroscopic adhesiolysis: a randomized controlled trial. Reprod BioMed Online. (2022) 45:1189–96. doi: 10.1016/j.rbmo.2022.07.003 36184275

[33] Tang R Xiao X He Y Qiu D Zhang W Wang X . Clinical evaluation of autologous platelet-rich plasma therapy for intrauterine adhesions: a systematic review and meta-analysis. Front Endocrinol (Lausanne). (2023) 14:1183209. doi: 10.3389/fendo.2023.1183209 37484965 PMC10359885

