# Supplementary material for: Effect of magnetic thermal vibration therapy combined with Femoston on endometrial repair in postoperative patients with moderate to severe intrauterine adhesions: a randomized controlled trial
Source: Front Endocrinol (Lausanne). 2026 Aug 26;17:1851945. doi: 10.3389/fendo.2026.1851945 (PMC13553263; doi:10.3389/fendo.2026.1851945)
Supplement: Supplementary file 1 [file Table1.docx]

**Supplementary Table S1.** Evidence supporting the plausibility and standardization of the magnetic thermal vibration therapy regimen used in this trial

| **Parameter** | **Trial regimen** | **Manufacturer information** | **Literature** | **Reference** |
| --- | --- | --- | --- | --- |
| Device-class standardization | CCRZR31 magnetic thermal vibration therapy unit | The public technical page for the CCRZR31 lists a device class combining magnetic field, vibration, and heat, with temperature presets of 40°C, 46°C, 52°C, and 58°C, vibration frequency 50 Hz ± 1, vibration range 2–5 mm, and treatment time 0–99 min. | Similar magnetic vibration and heat therapy devices from the same device family also list the same preset structure. | (1-3) |
| Temperature setting | 46°C | Across the exact model page and similar device-family pages, 46°C repeatedly appears as one of the manufacturer-defined preset temperature levels within a broader adjustable range of 40–58°C. |  | (1) |
| Vibration frequency | 50 Hz | The CCRZR31 and comparable magnetic vibration/heat therapy devices repeatedly specify 50 Hz ± 1 as the standard vibration frequency. | In adjacent microcirculation literature, local vibration frequencies in the 20–50 Hz range have been associated with improved blood microcirculation, and 50 Hz has been reported to produce strong skin-blood-flow responses in several local-vibration studies. | (1-3) |
| Magnetic field intensity | 50 mT | Broader procurement and technical-specification documents for magnetic vibration heat therapy devices often describe magnetic induction adjustable in the 10–50 mT range and vibration options of 30/40/50/60 Hz. |  | (4-6) |
| Session duration | 30 min/session | The public CCRZR31 page lists 0–99 min adjustable treatment time. | Procurement specifications for similar devices describe treatment times that are adjustable and can extend to ≥60 min. | (1-3) |
| Treatment course | Every other day for 20 days; total 10 sessions | The manuscript protocol reflects a standardized institutional regimen applied uniformly to all treated participants. | We did not identify published IUA-specific parameter-optimization trials defining the optimal number of sessions or exact course for magnetic thermal vibration therapy after adhesiolysis. |  |
| Vibration-related microcirculation rationale | 50 Hz local vibration |  | A Frontiers study on local vibration reported that vibration interventions in the 20–50 Hz range were beneficial to blood microcirculation and that 50 Hz may produce higher increases in skin blood flow; the same paper cites prior work showing 30 Hz and 50 Hz local vibration both increased skin blood flow, with some studies favoring 50 Hz. | (7) |

**Abbreviations**: IUA, intrauterine adhesions; PBAC, Pictorial Blood Loss Assessment Chart.

1- China Care Medical Equipment. Magnetic Vibration and Heat Therapy Unit (CCRZR31): technical specifications. Available from the manufacturer product page at: <https://www.chinacaremedical.com/magnetic-vibration-and-heating_p2267.html>
2- Sunnyou Medical. Magnetic Vibration and Heat Therapy Device XY-K-CZR-I: parameters. Available from manufacturer product page at: <https://www.sunnyou.com/product/82.html>
3- Sunnyou Medical. Magnetic Vibration and Heat Therapy Device XY-K-CZR-III: parameters and product advantages. Available from manufacturer product page at: <https://www.sunnyou.com/product/81.html>
4- Lu’an People’s Hospital / procurement specification document at: <https://www.laey.net/uploadfiles/2020/01/202001091628302830.doc>
5- Blue Tian County People’s Hospital procurement specification at: <https://www.ccgp-shaanxi.gov.cn/gpx-bid-file/ZF_JGBM_000003/zone/2025/1/12/project/gpx-template/8a69c77594582cc70194e4d61069546e.pdf?accessCode=e74ecbd2d6422531e4f92443793ffef5>
6- Qingdao procurement specification document. Magnetic vibration heat therapy device, including magnetic intensity 10–50 mT and vibration frequencies 30/40/50/60 Hz available at: <https://ggzy.qingdao.gov.cn/PortalQDManage/PortalQD/GetZbDownLoad?filePOrB=2&id=90339>
7- Ren W, Pu F, Luan H, Duan Y, Su H, Fan Y and Jan Y-K (2019) Effects of Local Vibration With Different Intermittent Durations on Skin Blood Flow Responses in Diabetic People. Front. Bioeng. Biotechnol. 7:310. doi: 10.3389/fbioe.2019.00310
8- Kim, HS., Baby, T., Lee, JH. et al. Biomaterials-enabled electrical stimulation for tissue healing and regeneration. Med-X 2, 7 (2024). <https://doi.org/10.1007/s44258-024-00020-8>
